# An electropneumatic cleaning device for piezo-actuator-driven picolitre-droplet dispensers

**DOI:** 10.1107/S1600576723009573

**Published:** 2024-02-01

**Authors:** Alexander Berkes, Stephan Kleine-Doepke, Jan-Philipp Leimkohl, Hendrik Schikora, Pedram Mehrabi, Friedjof Tellkamp, Eike C. Schulz

**Affiliations:** a Institute for Nanostructure and Solid State Physics, University of Hamburg, Hamburg, Germany; b Max-Planck-Institute for the Structure and Dynamics of Matter, Hamburg, Germany; c University Medical Center Hamburg-Eppendorf (UKE), Hamburg, Germany; SLAC National Accelerator Laboratory, Menlo Park, USA

**Keywords:** droplet deposition, serial crystallography, piezo-actuators, time-resolved crystallography

## Abstract

Recently, we introduced the liquid application method for time-resolved analyses (LAMA). The time-consuming cleaning cycles required for the substrate solution exchange and storage of the sensitive droplet-dispenser nozzles present practical challenges. In this work, a dispenser cleaning system for the semi-automated cleaning of the piezo-actuator-driven picolitre-droplet dispensers required for LAMA is introduced to streamline typical workflows.

## Abstract 2

A device for automated cleaning of piezo-actuator-driven picolitre-droplet dispensers required for the liquid application method for time-resolved analyses is presented.

## 1. Introduction

Time-resolved X-ray crystallography (TRX) is the method of choice when it comes to obtaining a detailed understanding of the molecular mechanisms of proteins (Schulz *et al.*, 2022 ▸; Wilson, 2022 ▸). It has been demonstrated to provide key insights into reaction intermediates of enzymatic reactions, which are important in rationalizing structure–function relationships (Mehrabi *et al.*, 2019*b* ▸). While TRX is traditionally carried out on single crystals, the advent of X-ray free-electron laser (XFEL) sources has triggered a number of serial data collection approaches which have become the standard choice when carrying out these experiments at XFELs and synchrotrons alike (Chapman, 2019 ▸; Orville, 2020 ▸; Pearson & Mehrabi, 2020 ▸; Mehrabi *et al.*, 2021 ▸). In addition to optical excitation methods, *in situ* mixing has become an important approach for reaction initiation in TRX experiments (Chapman, 2019 ▸; Schulz *et al.*, 2022 ▸; Barends *et al.*, 2022 ▸). Serial crystallography typically relies on protein crystals with micrometre dimensions, which permit small-molecule diffusion times in the millisecond time domain (Schmidt, 2013 ▸, 2020 ▸; Mehrabi *et al.*, 2019*a* ▸; Butryn *et al.*, 2021 ▸; Beyerlein *et al.*, 2017 ▸). Considering that the median turnover time of most enzymes lies within this time regime, the practical simplicity and wide applicability make *in situ* mixing methods an attractive option for a large number of systems (Bar-Even *et al.*, 2011 ▸). Focusing on liquid jet systems, a variety of T-junction mixing devices have been developed that commonly mix a suspension of micro-crystals with a substrate solution; the flow speed and the distance from the mixing point to the point of interaction with the X-rays define the delay time (Stagno *et al.*, 2017 ▸; Wilson, 2022 ▸). An alternative *in situ* mixing approach is adopted by tape-drive delivery systems; here droplets of substrate solution are deposited onto droplets of crystal solution (Beyerlein *et al.*, 2017 ▸; Wilson, 2022 ▸). Similarly to the T-junction mixers, the speed of the tape drive and the distance from the mixing point to the point of interaction with the X-rays define the delay time (Stagno *et al.*, 2017 ▸; Beyerlein *et al.*, 2017 ▸; Wilson, 2022 ▸).

To enable *in situ* mixing with fixed targets we developed a different solution and have recently introduced the liquid application method for time-resolved analysis (LAMA) (Mehrabi *et al.*, 2019*a* ▸), enabling reaction initiation in fixed-target serial crystallography applications with the hit-and-return chip and the Spitrobot crystal plunger (Mehrabi *et al.*, 2020 ▸, 2023 ▸; Schulz *et al.*, 2018 ▸). The general working principle of LAMA relies on 75–150 pl droplets of substrate solution, which are ejected via piezo-actuators from 50–70 µm dispenser openings onto protein crystals. For serial data collections, the motion of the fixed-target chips brings the crystals to the X-ray interaction point after a defined delay time (Schulz *et al.*, 2018 ▸). If used in combination with the Spitrobot crystal plunger, the crystals are vitrified in liquid nitrogen after a pre-defined delay time after droplet deposition (Mehrabi *et al.*, 2023 ▸). As with other liquid-handling methods, cleanliness of the dispenser nozzles is a general requirement to maintain chemical rigour between different substrate solutions and to avoid clogging of the dispensers during storage. In practice, cleaning the tubing and micro-capillaries of the LAMA system can be time consuming. Making use of the built-in purging protocol provided by the manufacturer requires multiple cycles of complete filling and draining of the dispenser. As each cycle is associated with a system-dependent waiting period, swapping substrate solutions between different crystal samples can substantially delay progress during beam time. Thus we sought a robust and efficient way to clean the LAMA dispenser nozzle that would avoid any cross-contamination between applications and extend the nozzle lifetime with minimal user interaction. To this end, we have developed an electropneumatic cleaning device (the ‘dispenser cleaning system’) that automatically aspirates and drains the LAMA dispenser nozzles with a cleaning solution of choice. This accessory device enables swift nozzle cleaning between swapping substrate solution or prior to nozzle storage with minimal user interaction.

## 2. Materials and methods

### 2.1. The dispenser cleaning system

**Figure 1:**
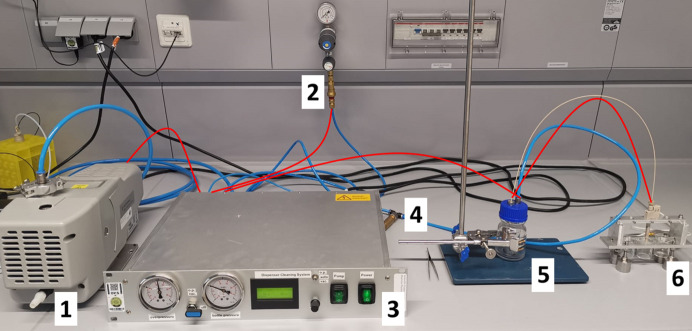
Laboratory setup for the dispenser cleaning system: (1) a vacuum pump, (2) connection to compressed air, (3) a control unit, (4) a pressure-relief valve, (5) a 100 ml reservoir bottle containing cleaning solution and (6) a nozzle holder connected to a 10 ml waste container. Pneumatic connections between parts of the machine are indicated by red lines.

The dispenser cleaning system consists of a dry scroll vacuum pump (IDP-3, Agilent Technologies, Santa Clara, USA), a control unit, a reservoir comprising an over-pressure bottle with a 10 ml container (Duran GL 45, DWK Life Sciences, Wertheim, Germany) and a holder for LAMA dispenser nozzles (Fig. 1 ▸). All information required to reproduce the control unit and the dispenser nozzle holder is provided in the supporting information.

#### 2.1.1. Control unit

**Figure 2:**
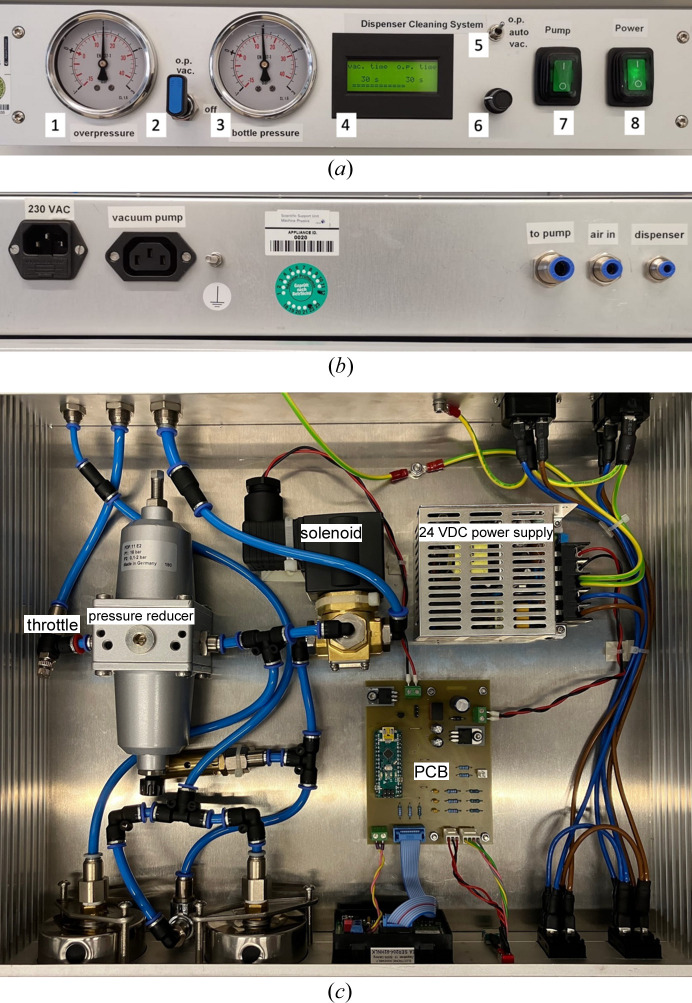
Control unit of the dispenser cleaning system: (*a*) front, (*b*) back and (*c*) internal views. (1) Over-pressure gauge, (2) vacuum control switch, (3) bottle-pressure gauge, (4) cleaning cycle display, (5) cleaning mode control switch, (6) duty cycle dial, (7) pump power switch, (8) main power switch.

For user convenience, all parameters are controlled via a central control unit, to which compressed air and the vacuum pump are connected. Within the control unit, two analog pressure gauges display the pressure in the dispenser cleaning system (Fig. 2 ▸). The pneumatic connection from the control unit to the reservoir bottle can be regulated by a valve. Via a control switch, three operational modes can be set: (1) over-pressure (‘o.p.’), (2) under-pressure (‘vac.’) and (3) alternating (‘auto’).

In auto-mode the time intervals for applying over- and under-pressure can be electronically set between 1 and 300 s. A digital display shows the set interval time and the elapsed time of the cycle. The back of the control unit bears the electric and pneumatic connections. The vacuum pump is powered via a feed-through in the control unit, controlled by a switch next to the power switch for the control unit itself.

#### 2.1.2. The reservoir

**Figure 3:**
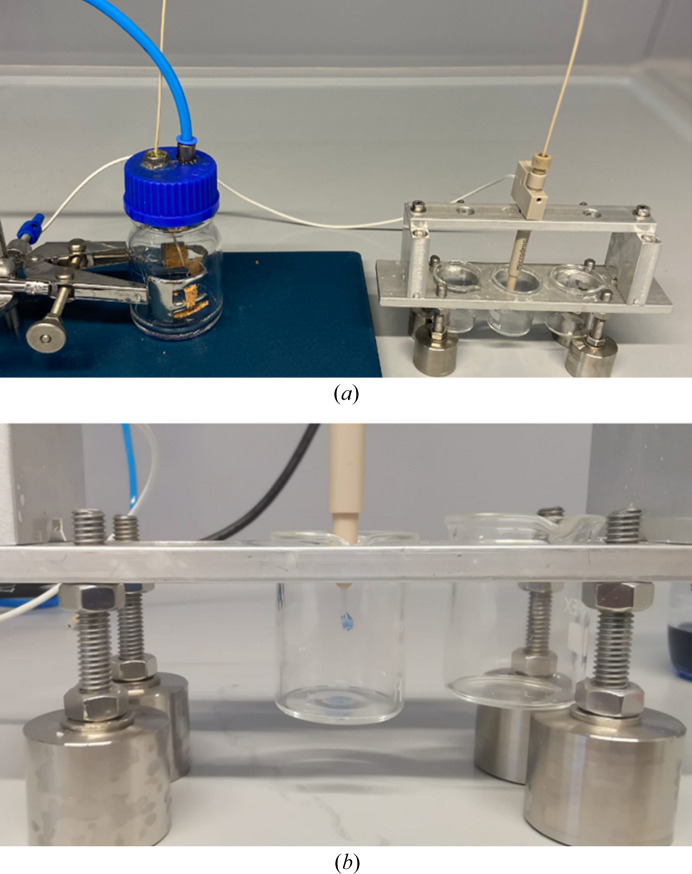
(*a*) Reservoir bottle connected to the LAMA droplet dispenser nozzle. (*b*) Close-up of the dispenser nozzle holder over the waste container while a droplet of the blue model contaminant is being pushed out.

The control unit is connected to the reservoir bottle via the pressure-relief valve. This valve limits the maximum pressure to 1 bar to prevent damage to the dispenser nozzles. The cleaning solution can be placed either directly within the reservoir bottle or alternatively within a 10 ml container inside the reservoir bottle. The reservoir bottle is airtight so that pressure is only exchanged via the nozzle, which is mounted in a specially designed nozzle holder above the waste container (Fig. 3 ▸).

### 2.2. Model contaminants

To test the cleaning ability of the dispenser cleaning system, it was compared with the manufacturers microdrop controller dispensing system (MD-E-3000; Microdrop Technologies, Norderstedt, Germany), which is used to control regular operation of the piezo-actuator droplet dispensers. In order to monitor cleaning success, we employed four standardized colour solutions as ‘model contaminants’, approximating typical crystallization solutions: 0.1%(*w*/*v*) coomassie brilliant blue in (*a*) 60 m*M* Tris pH 7.4, (*b*) 30%(*v*/*v*) 2-methyl-2,4-pentanediol (MPD), (*c*) 15%(*w*/*v*) PEG 3350 and (*d*) 1 *M* (NH_4_)_2_SO_4_, 60 m*M* Tris pH 7.4. Absorption was determined spectroscopically at 595 nm (NanoDrop 2000 ▸, ThermoFisher Scientific) at 1 s intervals during the first 10 s and at 20, 50, 100 and 180 s during the cleaning process with double-distilled water (ddH_2_O), as well as during subsequent cleaning with 2-propanol. All solutions, with the exception of the 1 *M* ammonium sulfate model contaminant, were filtered through a 0.45 m filter before use. Filtering the 1 *M* (NH_4_)_2_SO_4_ resulted in a colourless solution, and thus the solution was centrifuged at 3500*g* for 1 min.

## 3. Results and discussion

Owing to its simplicity and applicability to a wide number of target systems, *in situ* mixing is emerging as a convenient method for reaction initiation in TRX experiments. LAMA was demonstrated to be a versatile approach, not only with respect to achievable time delays and different substrate solutions but also regarding completely different experimental setups (Mehrabi *et al.*, 2019*a* ▸, 2023 ▸). As more users carry out these experiments, both the variety and the number of different substrate solutions sampled increase. Switching between different substrate solutions requires adhering to chemical cleanliness and hence demands thorough cleaning of the dispenser nozzles. Therefore, any given substrate solution has to be replaced with ddH_2_O and then air prior to storage. Moreover, the substrate solutions typically sprayed from LAMA droplet dispensers aim to closely match the environment of the crystals to avoid any unnecessary change in their surroundings. For Spitrobot experiments, the solutions are further supplemented with cryo-protectants (Mehrabi *et al.*, 2023 ▸). This, and the large variability of crystallization solution compositions, usually require testing and optimization of droplet-formation conditions. Although some general guidelines can be followed when preparing new substrate solutions (*e.g.* not exceeding viscosity limits, careful sterile filtration *etc.*), it is not unusual that, during these initial optimization trials, clogged nozzles can be encountered, which require careful cleaning before the next iteration. The microdrop controller provided by the manufacturer can apply uninter­rupted over- or under-pressure of up to 750 mbar for up to 50 s. However, the system can not provide alternating cycles, nor can the run time be extended. A major practical challenge is also that after each cycle the system has to re-equilibrate to ambient pressure, which takes around 15 s. Thus, in order to achieve sufficiently clean dispenser nozzles the microdrop controller is blocked for several minutes and requires continuous user supervision. This reduces effective progress during beam time, in particular when reaction initiation with a variety of substrate solutions has to be tested. In contrast, our dispenser cleaning system can continuously apply up to 1 bar of over-pressure or under-pressure to a fluid (liquid or air) which is either pushed or pulled through a droplet dispenser nozzle (Fig. 1 ▸). Over-pressure is realized via a connection to compressed air in the laboratory, and under-pressure is realized via a vacuum pump. Alternating repetitive cycles of aspiration and draining clean the droplet dispensers over time. A cleaning solution, typically either ddH_2_O or 2-propanol (see below), is provided in the reservoir directly connected to the dispenser nozzle. By applying over-pressure to this reservoir, the fluid is pushed through the dispenser nozzle; conversely, applying under-pressure aspirates the fluid.

### 3.1. Dispenser cleaning system protocol

For a general cleaning protocol, we sought common laboratory consumables that are generally available and safe to use. The dispenser nozzles are made of glass, and thus harsh cleaning reagents such as acids and bases (*e.g.* HCl or NaOH) offer a viable cleaning option. However, for a standard cleaning protocol we opted for milder substances. After an exchange of substrate solutions or prior to storage of the dispenser nozzles, the dispenser nozzles are cleaned by initially pushing sterile filtered ddH_2_O followed by sterile filtered 2-propanol through for 3 min each. The primary purpose of the 2-propanol step is to replace water prior to the final drying step as it evaporates faster and thus improves the drying process. Finally, a 5 min-long drying step concludes the cleaning protocol, during which air is repeatedly pulled and pushed through the nozzle for 30 s each. To enable a direct comparison, we carried out the cleaning protocol using the dispenser cleaning system, the microdrop controller and a 50 ml syringe for manual cleaning. The results are summarized in Table 1 ▸. In a direct comparison, the primary cleaning protocol can be carried out approximately 15–20% faster by the dispenser cleaning system than via the microdrop controller. While this gain is probably limited, the primary advantage lies in the reduced user interventions required for each cleaning step. For manual cleaning via a syringe, we note (*a*) the substantial physical strength required to push the solutions through the nozzle and (*b*) the reduced flow rate. The dispenser cleaning system and the microdrop controller achieve a flow rate of approximately 0.9 ml min^−1^, whereas manual syringe cleaning can only achieve 0.2–0.4 ml min^−1^ depending on user strength. Thus during the same cleaning time, less than half of the cleaning solution can be pushed through the nozzles via manual cleaning.

### 3.2. Cleaning performance

**Figure 4:**
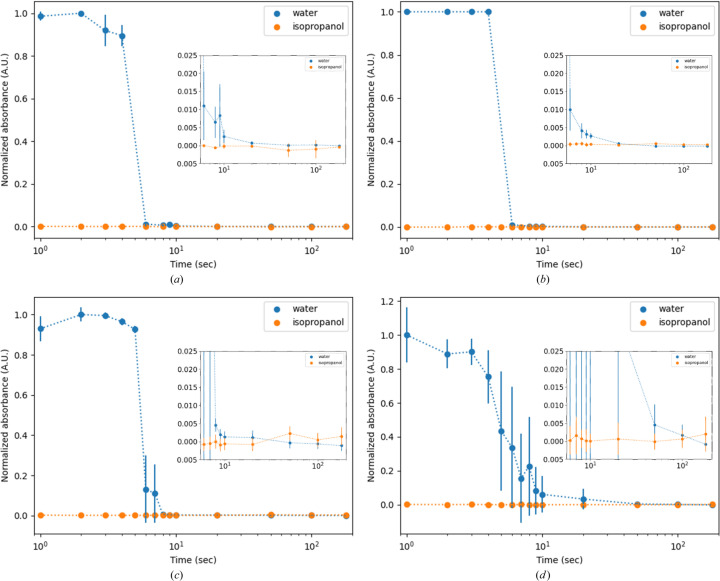
Normalized absorbance at 595 nm during the cleaning process. Cleaning performance using the model contaminants. The inset shows the area between 6 and 180 s. (*a*) Buffer, (*b*) 30% MPD, (*c*) 15%(*w*/*v*) PEG3350, (*d*) 1 *M* (NH_4_)_2_SO_4_. For (*a*)–(*c*) *n* = 3 and for (*d*) *n* = 6. Error bars represent the standard deviation.

To test the effectiveness of our cleaning protocol, we utilized four different model contaminants, three of which also approximate typical crystallization solutions based on MPD, PEG and (NH_4_)_2_SO_4_. The model contaminants were aspirated for 30 s into the nozzle via the microdrop controller. Cleaning success was monitored via absorbance at 595 nm (Fig. 4 ▸). For each model contaminant, absorption was recorded at 1 s intervals during the first 10 s and at 20, 50, 100 and 180 s during the cleaning process with ddH_2_O as well as during the subsequent rinsing with 2-pro­panol. Each measurement was normalized to a maximum absorption of 1. The procedure was repeated 3–6 times for each model contaminant.

Cleaning success is demonstrated by a reduction in 595 nm absorbance, which returns to baseline within roughly 10 s for each model contaminant. However, it is also obvious that, depending on the precipitant present in the model contaminant, reduction to baseline absorbance varies substantially. The water- and MPD-based solutions behave reproducibly, whereas the PEG- and (NH_4_)_2_SO_4_-based solutions show higher variability, presumably due to differences in viscosity and miscibility with the cleaning solutions. This matches practical experience that more viscous solutions typically require more stringent cleaning cycles. In particular, the latter situations are improved by employing the dispenser cleaning system, which enables thorough dispenser nozzle cleaning, largely without user intervention, and liberates the microdrop controller for additional LAMA experiments, provided additional dispenser nozzles are available.

### 3.3. Nozzle declogging with the dispenser cleaning system

The main advantage of the dispenser cleaning system lies in its ability to effectively clear clogged nozzles. Typically, clogging occurs during initial testing or, for example, due to crystallization of highly concentrated substrate solutions by inadvertent drying of the nozzle. The dispenser cleaning system can alternate between applying 1 bar of over- and under-pressure while the tip of the dispenser nozzle is also submerged in cleaning reagent, such that an alternating forward and backward flow is achieved. Under these circumstances, it has proven helpful to make use of more the rigorous cleaning reagents mentioned above (*e.g.* HCl or NaOH) or mild detergent solutions [*e.g.* 1%(*w*/*v*) Tergazyme or Hellmanex]. In addition, declogging is generally improved when using warm solutions and by activating the piezo-actuator cuff via the microdrop controller and operating it at a frequency of up to 5 kHz during the declogging protocol. Although this occupies the microdrop controller and prevents its use at, for example, the beamline, the dispenser cleaning system can be operated with minimal user intervention. Thus a declogging procedure can be started and left to run for several hours (*e.g.* overnight) when the microdrop controller is not in use. Although manual cleaning can also remove a clog, it is associated with an increased risk of both damaging the nozzles and harming the user as high pressure must be applied to liberate a clog. The microdrop controller, on the other hand, only applies a pressure of 750 mbar for no more than 50 s at a time, which in our experience is usually ineffective for successfully removing a clog. Moreover, unlike the dispenser cleaning system it is unable to automatically change the direction of flow, a strategy that has proven very helpful in removing clogs. After the clog has been removed the standard cleaning protocol should be applied.

## 4. Conclusion

We have found the dispenser cleaning system to be a helpful accessory in day-to-day operation of LAMA dispenser nozzles, which have become an irreplaceable asset in our TRX experiments.

## Supplementary Material

Supporting information, including a readme describing its contents. DOI: 10.1107/S1600576723009573/te5126sup1.zip

## Figures and Tables

**Table 1:** Comparison of the LAMA nozzle cleaning protocol using different setups

	Dispenser cleaning system			MicroDrop controller			Manual syringe cleaning		
	User interventions	Time (s)	Volume (ml)	User interventions	Time (s)	Volume (ml)	User interventions	Time (s)	Volume (ml)
Exchange of solution	1	60		1	60		1	60	
Rinse with ddH_2_O	1	180	2.7	5	215†	2.7	1	180	0.9§
Exchange of solution	1	60		1	60		1	60	
Rinse with 2-propanol	1	180	2.7	5	215†	2.7	1	180	0.9§
Exchange to air	1	60		1	60		1	60	
5 min air drying	1	300		6	390‡		1	300	
Total	6	840	5.4	19	1000	5.4	6	840	1.8

† Requires three restarts of 15 s each.

‡ Requires six restarts of 15 s each.

§ Reduced flow rate.
