# Supplementary figures and images for: An electropneumatic cleaning device for piezo-actuator-driven picolitre-droplet dispensers
Source: J Appl Crystallogr. 2024 Feb 1;57(Pt 1):209–14. doi: 10.1107/S1600576723009573 (PMC10840313; doi:10.1107/S1600576723009573)

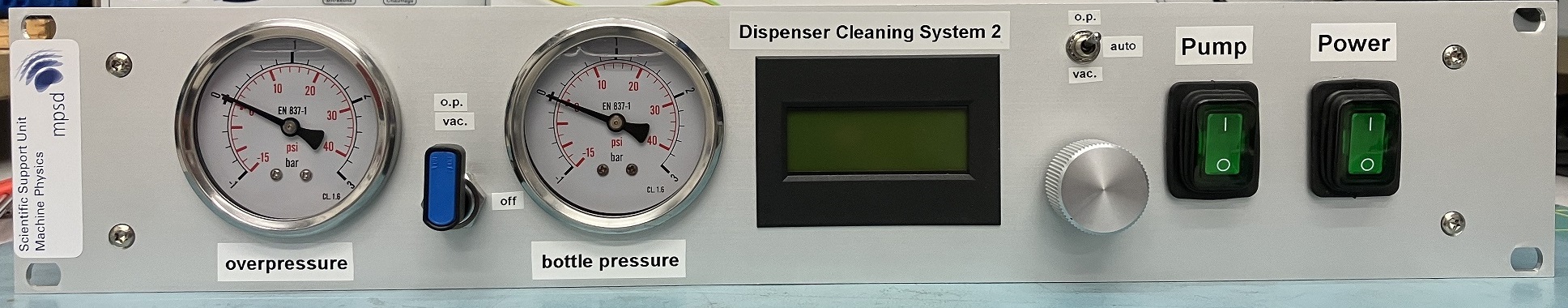

Supplement: Supplementary file 1 [file j-57-00209-sup1.zip › CM doc/images/front_view.jpg]

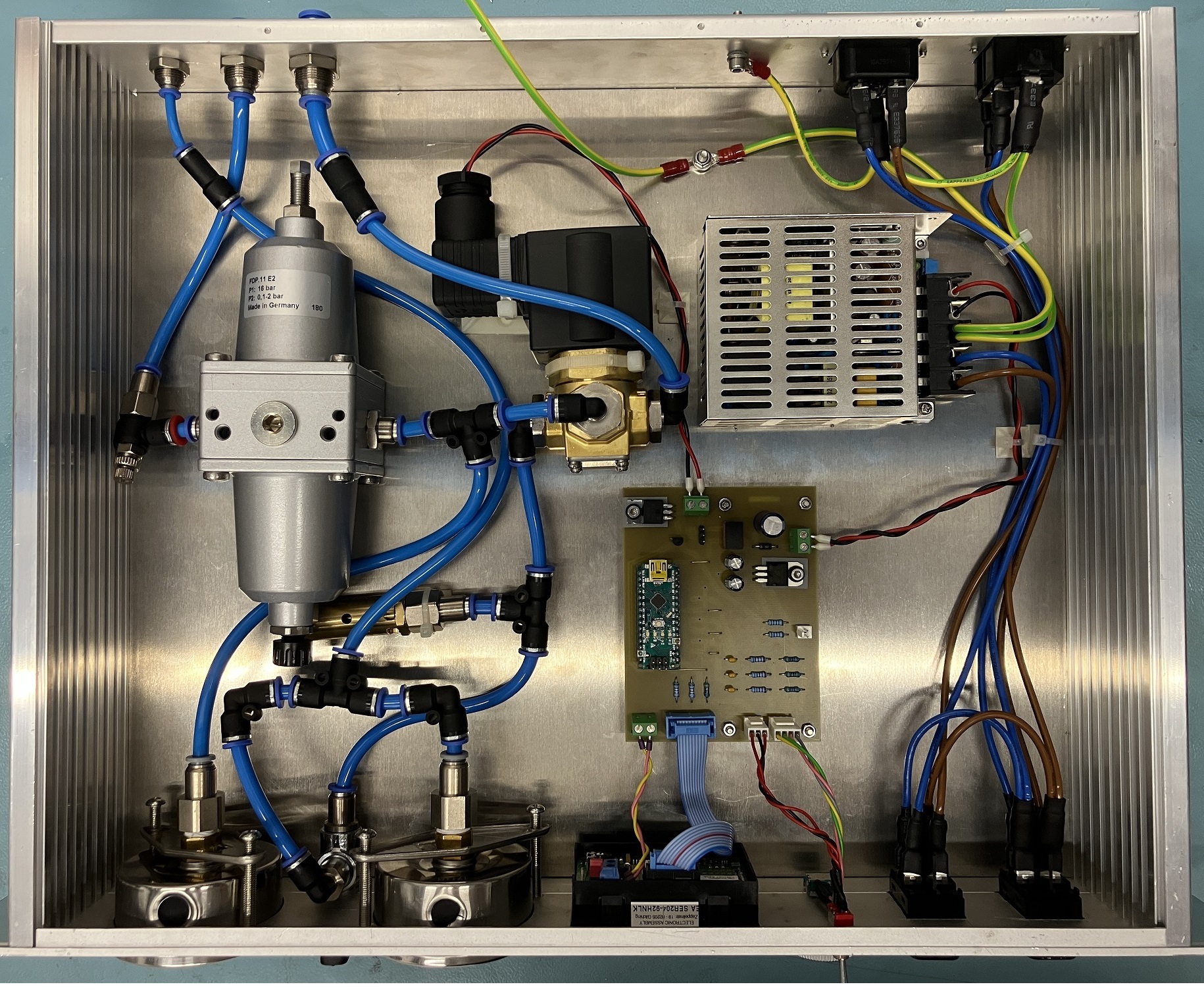

Supplement: Supplementary file 1 [file j-57-00209-sup1.zip › CM doc/images/top_view.jpg]

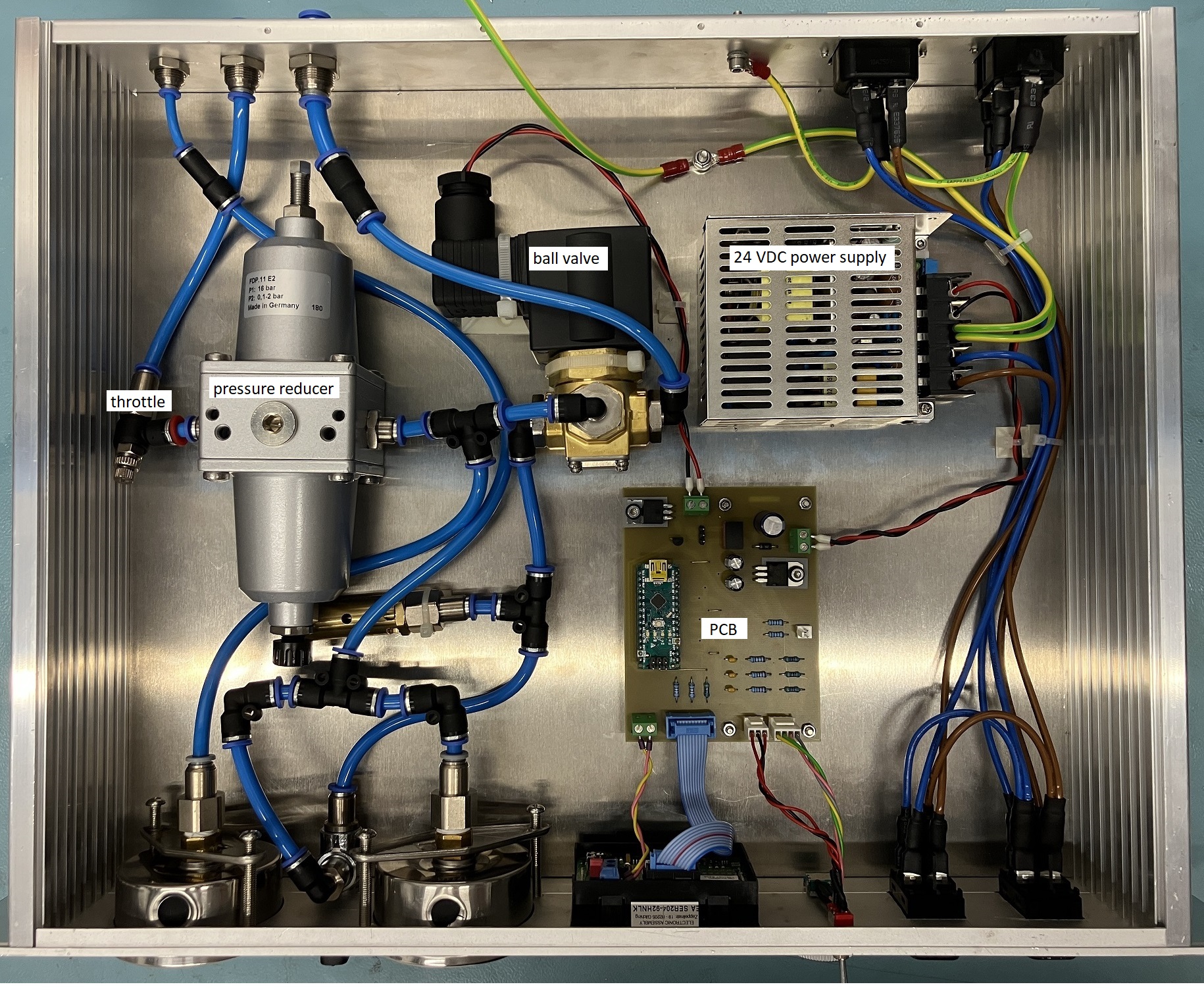

Supplement: Supplementary file 1 [file j-57-00209-sup1.zip › CM doc/images/top_view_label.jpg]

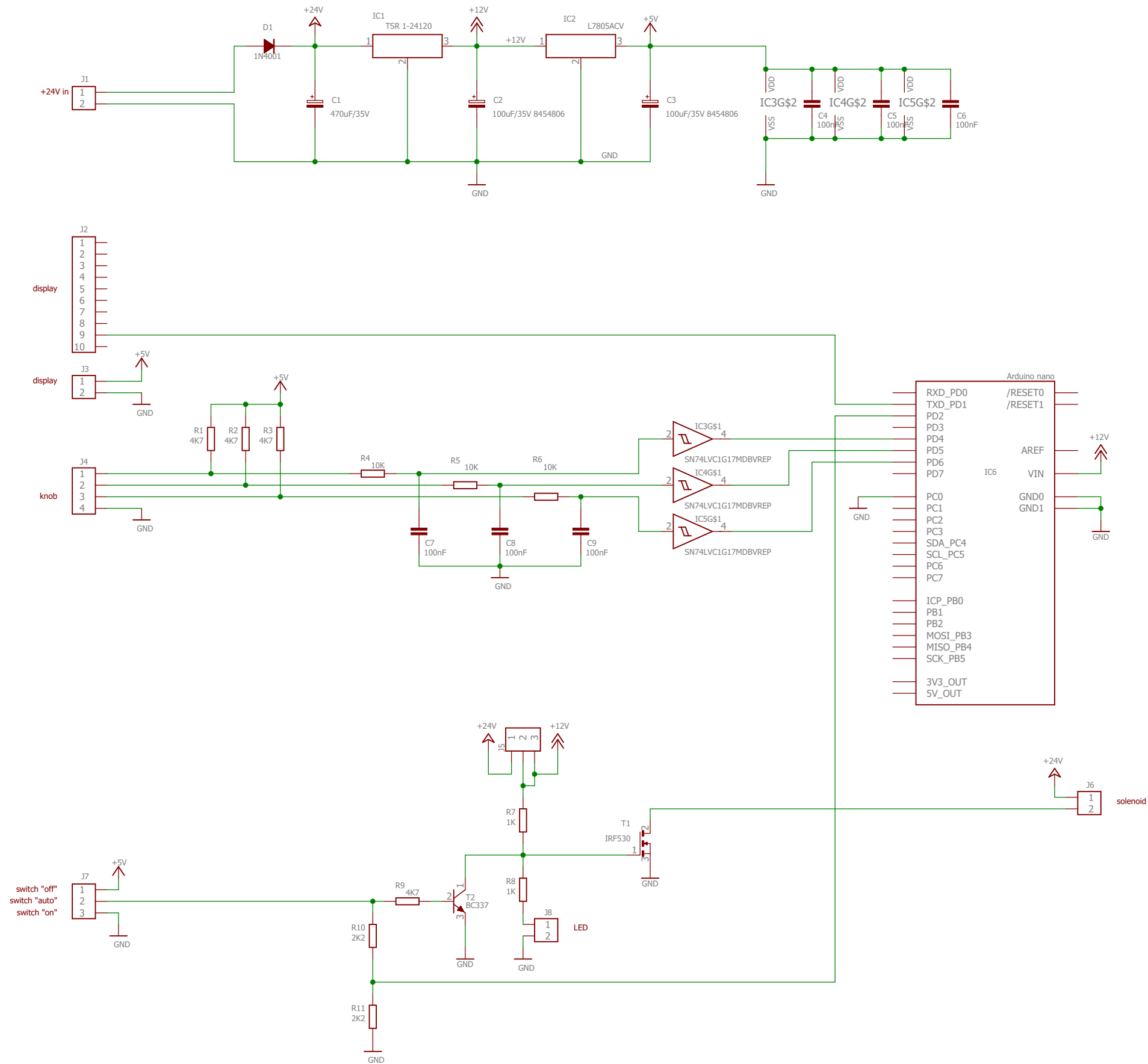

Supplement: Supplementary file 1 [file j-57-00209-sup1.zip › CM doc/electronics/LMK220152-sch.pdf]
